# Maximising value from a United Kingdom Biomedical Research Centre: study protocol

**DOI:** 10.1186/s12961-017-0237-1

**Published:** 2017-08-14

**Authors:** Trisha Greenhalgh, Pavel V. Ovseiko, Nick Fahy, Sara Shaw, Polly Kerr, Alexander D. Rushforth, Keith M. Channon, Vasiliki Kiparoglou, On behalf of the Partnerships for Health, Wealth and Innovation cross-cutting theme of the National Institute for Health Research Biomedical Research Centre, Oxford, Maxine Allen, Jeroen Bergmann, Denise Best, Jacqueline Birks, Chas Bountra, Alastair Buchan, Gary Collins, Stuart Faulkner, Gary Ford, John Geddes, Alastair Gray, Louise Locock, Alison Noble, Christopher Pugh, Mark Sheehan, Joel Smith, Adam Stoten, Charles Vincent, Glenn Wells, Paul Whyte

**Affiliations:** 1 Nuffield Department of Primary Care Health Sciences, University of Oxford, Radcliffe Primary Care Building, Woodstock Rd, Oxford, OX2 6GG United Kingdom; 2 Radcliffe Department of Medicine, University of Oxford, John Radcliffe Hospital, Oxford, OX3 9DU United Kingdom; 3 Oxford University Hospitals NHS Foundation Trust, John Radcliffe Hospital, Oxford, OX3 9DU United Kingdom

**Keywords:** Innovation policy, Health policy, Health research policy, Health technology development, National Institute for Health Research, Biomedical Research Centres, Research partnerships, Research on research, Knowledge production

## Abstract

**Background:**

Biomedical Research Centres (BRCs) are partnerships between healthcare organisations and universities in England. Their mission is to generate novel treatments, technologies, diagnostics and other interventions that increase the country’s international competitiveness, to rapidly translate these innovations into benefits for patients, and to improve efficiency and reduce waste in healthcare. As NIHR Oxford BRC (Oxford BRC) enters its third 5-year funding period, we seek to (1) apply the evidence base on how best to support the various partnerships in this large, multi-stakeholder research system and (2) research how these partnerships play out in a new, ambitious programme of translational research.

**Methods:**

Organisational case study, informed by the principles of action research. A cross-cutting theme, ‘Partnerships for Health, Wealth and Innovation’ has been established with multiple sub-themes (drug development, device development, business support and commercialisation, research methodology and statistics, health economics, bioethics, patient and public involvement and engagement, knowledge translation, and education and training) to support individual BRC research themes and generate cross-theme learning.

The ‘Partnerships’ theme will support the BRC’s goals by facilitating six types of partnership (with patients and citizens, clinical services, industry, across the NIHR infrastructure, across academic disciplines, and with policymakers and payers) through a range of engagement platforms and activities. We will develop a longitudinal progress narrative centred around exemplar case studies, and apply theoretical models from innovation studies (Triple Helix), sociology of science (Mode 2 knowledge production) and business studies (Value Co-creation). Data sources will be the empirical research studies within individual BRC research themes (who will apply separately for NHS ethics approval), plus documentary analysis and interviews and ethnography with research stakeholders. This study has received ethics clearance through the University of Oxford Central University Research Ethics Committee.

**Discussion:**

We anticipate that this work will add significant value to Oxford BRC. We predict accelerated knowledge translation; closer alignment of the innovation process with patient priorities and the principles of responsible, ethical research; reduction in research waste; new knowledge about the governance and activities of multi-stakeholder research partnerships and the contexts in which they operate; and capacity-building that reflects the future needs of a rapidly-evolving health research system.

## Background

Cross-sector research partnerships are an important component of the contemporary research landscape. The National Institute for Health Research (NIHR), for example, invests over £1 billion per year supporting partnerships between National Health Service (NHS) organisations and universities in England. Most notably, these include NIHR Biomedical Research Centres (BRCs) and NIHR Collaborations for Leadership in Applied Health Research and Care (CLAHRCs) [1–4]. The United Kingdom Department of Health also invests efforts and resources in wider multi-stakeholder partnerships, including Academic Health Science Centres (AHSCs) and Academic Health Science Networks [5–7], described as the NHS’s “*gateway to the life sciences industry*” [8]. All these structures encourage strong links with industry and high levels of patient and public involvement and engagement.

The goal of NIHR BRCs is to accelerate both ‘T1’ translational research (from the laboratory bench to clinical trials) and ‘T2’ translational research (from clinical trials to implementation in clinical practice and policy) [2, 9]. The first wave of these BRCs was established in 2007; as of April 2017, there are 20 such centres nationally. As stated by the NIHR, their aims are to [4] drive innovation in the prevention, diagnosis and treatment of ill-health, translate advances in biomedical research into benefits for patients, and help the NHS contribute to the nation’s international competitiveness.

Whilst NIHR BRCs are specific to the United Kingdom, the idea for cross-sector research partnerships aimed at rapidly promoting and translating life sciences and technology research into benefits for patients originated in the United States of America [10]. These networked structures are now a global phenomenon [11, 12], intended to generate synergies that drive innovation and accelerate the translational research pathway [3].

There is evidence to suggest that NIHR BRCs, including one in Oxford, have had some positive impacts on resource targeting, management and governance in translational research for patient benefit at the interface between NHS organisations, universities and industry, but there is also some evidence of negative impacts and a number of as yet unexploited opportunities to maximise the value of biomedical research [13–15]. Research on similar cross-sector translational research partnerships in other countries suggests that they sometimes, but not always, achieve their goals [16–18]. Some authors have argued that cross-sector partnerships in rapidly-developing, high-technology fields may generate substantial research waste as patients’ needs and priorities are overlooked in favour of the relentless pursuit of innovation and commercial influences that conflict with the pursuit of the public good [19–21]. Others have taken the opposite view and argued that there is evidence of significant societal impact and economies of scale from such partnerships [22]. A recent review that asked whether concentrating research funding into a small number of very large research centres (as in BRCs) was likely to produce economies or diseconomies of scale concluded that there were examples of both in the literature [23].

Underpinning the policy of ‘innovation, health and wealth’ is the implicit assumption that pursuit of the first of these goals (innovation) through what has been termed the ‘managed network’ approach [24] will – if strategic drivers are appropriately aligned – generate the second two [25–27]. The rationale for this policy is that, in order to keep pace with rising expectations and growing demand, especially when innovation generates interventions that are both effective and costly, the health sector needs to become more efficient, embrace innovations that are both effective and cost-effective, and generate additional income. Potentially, this can be achieved through a thriving regional innovation system that accelerates economic growth and wealth creation while at the same time generating worthwhile innovations for use in the NHS.

From its inception, the NIHR has strongly supported patient and public involvement and engagement (PPI/E), the former being defined as involvement of patients in the design, delivery and dissemination of research, and the latter as outreach from research scientists to communities and citizens [28]. The many and varied PPI/E activities by NIHR BRCs appear to align respectably with calls for the ‘democratisation’ of science [29, 30] and have produced some (albeit relatively weak) evidence of societal impact [31]. However, there is also evidence that the underpinning values driving high-technology biomedical research may sometimes be at odds with those of patients and citizens [32–34], and that, whilst BRC-funded researchers have been content for patients and the public to ‘tinker at the edges’ with science through consultation and outreach, some remain opposed to genuinely democratic partnerships in which citizens and scientists collaboratively set a research agenda and jointly oversee its delivery [35].

More broadly, BRCs lack an explicit link to the wider policy context for their work. NIHR BRCs are depicted as facilitating a pathway from basic bench science through to clinical practice, but – surprisingly – the surrounding policy context does not figure in core documents describing their scope and purpose [3, 9, 25]. Yet, this policy context heavily conditions that process of translation at every stage. It determines, for example, what other funds are available, what rules govern different stages of research such as clinical trials, what intellectual property rules apply to products versus service changes, and how different innovations relate to the financing and organisation of the NHS [4].

In sum, multi-stakeholder research partnerships, particularly in high-technology biomedical research with significant commercialisation potential, appear to be characterised by inherent complexity, multiple drivers, conflicting values and contested metrics of success. The potential synergies associated with these new organisational forms are considerable, but they are not guaranteed. Whilst the goals of ‘innovation’, ‘health’ and ‘wealth’ have been rhetorically aligned in policy documents and support a plausible narrative, they do not always march in step. Work must be done to maximise synergies, minimise waste and ensure responsible research and innovation.

Accordingly, as the NIHR Oxford BRC enters its third 5-year funding period (2017–2022), we present this study protocol for a programme of research seeking to (1) apply the existing evidence base on how best to support the various partnerships in this large, knowledge-based, multi-stakeholder research system; and (2) research how these partnerships play out in a new and ambitious programme of translational research.

### Rationale for this study protocol

Developing, pre-registering and publishing a study protocol for this novel programme of research spanning the entire NIHR Oxford BRC and other cross-sector research partnerships in the Oxford region is an important step towards maximising the value of the NIHR’s investment in research. It has the potential to strengthen the scientific rigour of the proposed research, optimise the efficiency of the research process, and improve the reproducibility of results, for the reasons stated below.

First, carefully developing and scrutinising research questions – both by the ‘Partnerships’ core research team and through independent oversight and external peer review – at the research design stage can simplify data collection, make data analysis more rigorous, and strengthen the quality of reporting [36]. We have developed this study protocol in collaboration with all researchers involved in the ‘Partnerships’ cross-cutting theme and have incorporated critical input from the leaders of other BRC themes and an external advisory group. To strengthen the quality of reporting and the potential for theorising, we will use published methodology for ‘n of 1’ case study research [37–39], adapting as appropriate to the unique nature of the case(s) under scrutiny.

Second, transparency can improve the quality of research and avoid duplication of effort by different researchers conducting similar research [40]. While registering experimental clinical studies on websites such as clinicaltrials.gov is now standard practice, this is not the case for qualitative case studies. To increase transparency and discoverability of our research, we have created a dedicated project page on a social networking site for scientists (see Dissemination section below). Moreover, to ensure unrestricted access to our research, we commit to publishing in open access journals.

Finally, publishing a study protocol helps mitigate publication bias, which (when it occurs) limits the available evidence base and wastes time and resources on repeating studies that have been conducted elsewhere. Large-scale organisational case studies of knowledge translation are rarely published unless they describe positive findings, which suggests that this study design may currently be particularly open to publication bias [41]. By publishing our study protocol, we make a public commitment to publishing all of our research, including both positive, negative and ambiguous findings.

## Setting and context

### Oxford regional innovation system

**Fig. 1:**
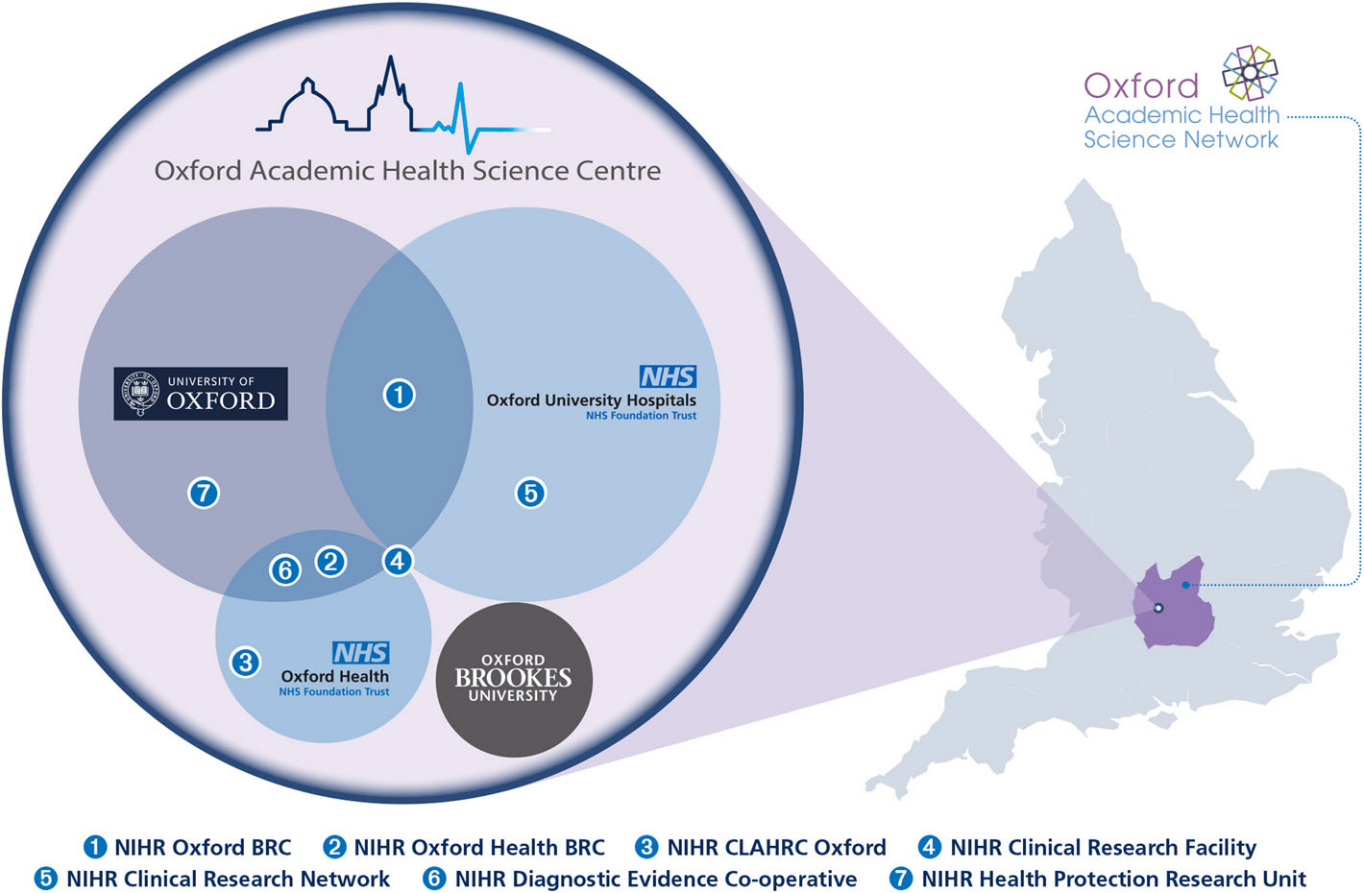
The Oxford regional innovation system for health, wealth and innovation: composition and geographical footprint (England). National Health Service (NHS), National Institute for Health Research (NIHR), Biomedical Research Centre (BRC), Collaboration for Leadership in Applied Health Research and Care (CLAHRC)

This study takes place in the Oxford region of the United Kingdom, which in addition to the NIHR Oxford BRC includes a comprehensive range of other NIHR research infrastructure within the AHSC in Oxford and the larger Academic Health Sciences Network region covering a population of 3.3 million across the Thames Valley and Milton Keynes (Fig. 1). The region is currently home to one of the world’s most significant biotechnology clusters and one of the United Kingdom’s leading NHS–university partnerships as measured by formal metrics of research volume, collaboration with industry, intellectual property and spin-out companies [42–45]. The region has a highly skilled labour force, especially in Oxfordshire, and over 46,000 jobs in high-tech firms across a range of technologies [43]. However, there are also pockets of deprivation and unemployment along with significant housing shortages and unmet health needs. A recent report on health inequalities in Oxfordshire highlighted the need for innovation, wealth creation and poverty reduction as part of a cross-sector approach to reducing inequalities [46].

### NIHR Oxford BRC

The NIHR Oxford BRC is based on partnerships between the Oxford University Hospitals NHS Foundation Trust and the University of Oxford, and externally with industry and other NIHR infrastructure (http://oxfordbrc.nihr.ac.uk). It was first established in 2007 with a NIHR award of £57 million over 5 years; renewed in 2012, with a NIHR award of £96 million for a further 5 years; and awarded £114 million of NIHR funding for 2017–2022. This funding buys out time of front-line clinicians and university academics and provides resources to conduct translational research across a range of disease-related and cross-cutting research themes. The NIHR Oxford BRC also played a seminal role in the development of a successful bid for a second BRC in Oxford, focusing on mental health and linked to the community-based Oxford Health NHS Foundation Trust. The NIHR Oxford Health BRC was established with a NIHR award of £13 million for 2017–2022. The two BRCs work closely together as part of Oxford AHSC in areas such as big data, personalised medicine, and multiple long-term conditions and dementia.

**Fig. 2:**
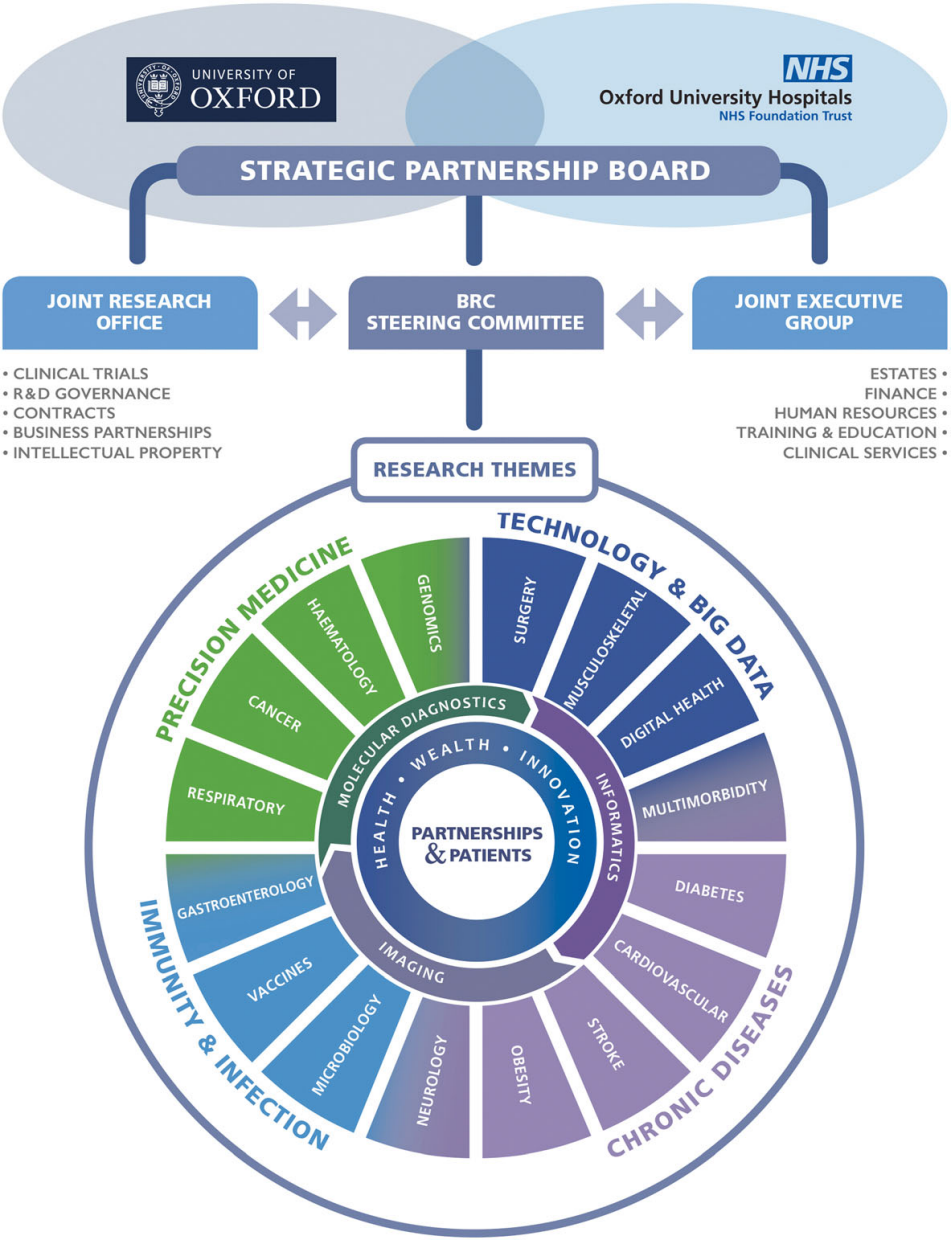
Structure and governance of NIHR Oxford BRC 2017–2022. The *lower circle* shows 16 research themes (spokes) grouped into four clusters (Chronic diseases, Precision medicine, Immunity and infection, and Technology and big data), along with cross-cutting themes (Molecular diagnostics, Informatics, Imaging, and Partnerships)

As of April 2017, the NIHR Oxford BRC has 16 disease-related research themes (e.g. diabetes, vaccines) organised into four clusters (Fig. 2). Each seeks to bring together scientific excellence, expertise and engagement platforms (see below for definition), thereby achieving focus and critical mass in a specific area. Grouping themes into clusters (e.g. chronic diseases) is a new approach from 2017, intended to amplify this critical mass and drive cross-disciplinary capabilities that address major healthcare opportunities and challenges such as prevention and early diagnosis. Also newly introduced in 2017 are formal cross-cutting themes (shown as the inner circular segments in Fig. 2) that provide technological platforms and generic capabilities (e.g. informatics, diagnostics) to support the disease-related research themes.

An important additional innovation for the NIHR Oxford BRC in 2017 has been the establishment of a large and integrative cross-cutting theme – ‘Partnerships for Health, Wealth and Innovation’, with multiple sub-themes (listed below under ‘Programme management and governance’). ‘Partnerships’, shown as the innermost circle in Fig. 2, aims to provide co-ordinated and targeted support to individual BRC themes and generate cross-theme learning through research on research. Below, we describe the aims, objectives, methodology, dissemination plan and intended outcomes of the Partnerships theme.

## Methods

### Aim of the NIHR Oxford BRC

The aim of the NIHR Oxford BRC 2017–2022 is to deliver on the NIHR’s stated objectives for BRCs (described in ‘Background’ above) through enhanced cross-disciplinary and inter-sectoral partnerships designed to produce synergies that can address major healthcare challenges at scale.

### Strategic objectives of the ‘Partnerships’ theme

**Fig. 3:**
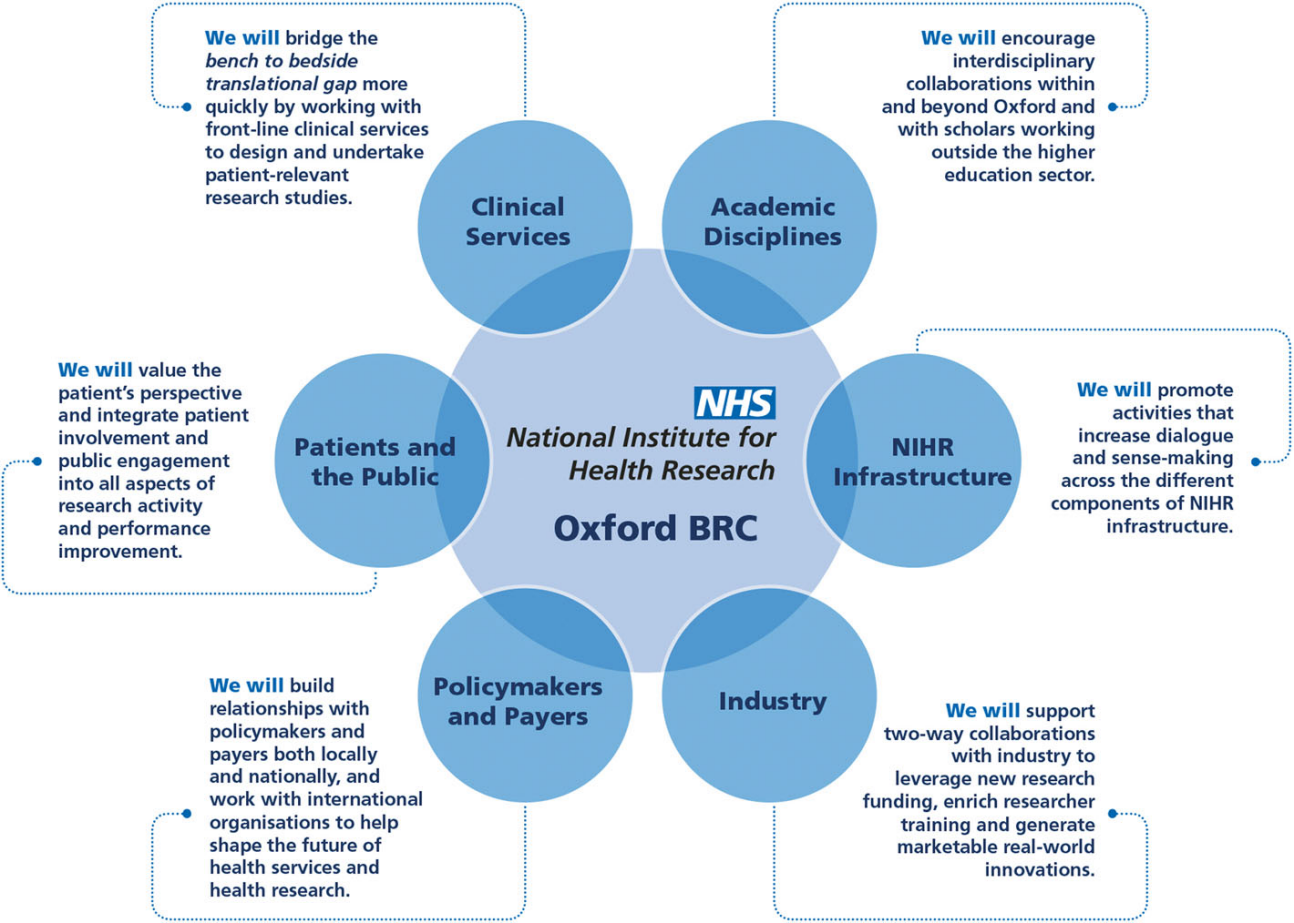
Key partnerships underpinning the NIHR Oxford Biomedical Research Centre, 2017–2022

The strategic objectives of the ‘Partnerships’ theme are (1) to support the aims of the NIHR Oxford BRC through a set of co-ordinated cross-cutting platforms and activities designed to strengthen six types of partnership (Fig. 3) and (2) to generate new knowledge about the governance and activities of high-technology, multi-stakeholder health research partnerships.

### Key partnerships underpinning the NIHR Oxford BRC 2017–2022

Figure 3 shows the key partnerships we seek to build. We expand on these below.

#### Partnerships with patients and the lay public to ensure relevance and accountability

NIHR Oxford BRC seeks to ensure that its research addresses patients’ priorities and is a good use of public funds. The ‘Partnerships’ external advisory group (see below) has been established with a lay chair and strong representation from patients and citizens. We will support all BRC research themes to value the patient’s perspective; integrate patient involvement and public engagement into all aspects of their research activity; measure their success using metrics that allow comparison over time and across themes; and improve their performance year on year.

#### Partnerships with clinical services to ensure rapid implementation of findings into practice

Traditionally, there has been an unacceptable delay between doing academic research and implementing research findings in a way that benefits patients. We know that, when research ideas come directly from front-line clinical practice and scientists work closely with clinicians to design and undertake research studies, this ‘bench to bedside translational gap’ can be bridged more effectively [47]. The ‘Partnerships’ theme will include a work stream on knowledge translation and implementation science (see these introductory papers for definitions and rationale [48, 49]) with a view to getting research discoveries rapidly into practice. Research links with clinical services that aim to introduce new drugs, devices or service models raise implications for local commissioning and funding models (see ‘Policymakers and payers’ below).

#### Partnerships with industry to maximise opportunities for innovation

The biotechnology, pharmaceuticals, medical devices and software industries are important contributors to both healthcare and national economic growth [34, 50]. We will help to link the BRC to industry partners with a view to building networks and collaborations that enable co-production of knowledge, leverage additional funding for research, enrich the training of early career researchers (e.g. through reciprocal placements) and increase economic and societal impact by helping to turn research ideas into marketable real-world innovations. We will also explore ways of making conflicts of interest explicit and ensuring that value for private-sector partners is not achieved at the expense of the public good.

#### Partnerships across the NIHR research infrastructure to ensure programme-wide learning

NIHR-funded research infrastructure in Oxford includes two BRCs, a CLAHRC, a Clinical Research Facility, a Clinical Research Network, a Health Protection Research Unit, and a Diagnostic Evidence Co-operative, which is currently being transformed into a MedTech and In Vitro Diagnostic Co-operative. This infrastructure embraces multiple research themes, theme clusters and cross-cutting themes, and covers all stages in the health research pathway from the basic sciences to implementation and evaluation of new service models. In large and complex research structures such as this, there is a danger that individual research teams could become isolated into silos and lose the sense of shared endeavour. By drawing out common challenges and emerging patterns across the Oxford BRC and beyond to Oxfordshire’s wider research infrastructure, we will help to engender a sense of coherence and generate knowledge that supports and improves the effectiveness of the NIHR infrastructure as a whole.

#### Partnerships across academic disciplines to develop new avenues of research

We will encourage interdisciplinary collaborations within the University of Oxford, with other higher education institutions within and beyond the United Kingdom, and with scholars who work outside the higher education sector. The BRC provides exciting opportunities for interdisciplinary collaborations between biomedical scientists and academics from complementary fields, including statistics, economics, computer science, business studies, and the humanities and social sciences. Interdisciplinary research enables large and ambitious projects (for example, multi-centre clinical trials or big data analytics). It also allows us to do research on research by asking over-arching questions about how to optimise the research process, improve the governance and ethics of research, train and support early career researchers, work with industry and government, and implement the findings of research in clinical practice.

#### Partnerships with policymakers and payers to shape the future of health services and health research

Biomedical research at Oxford seeks to improve the effectiveness and efficiency of local health services through evidence-based healthcare policymaking and commissioning and also to provide knowledge that can inform national and international-level decisions relating to research policy in the life sciences, technology and health sciences. The role of payers in bridging the translational gap between research and clinical services is critical, especially in times of financial austerity, but to date has been relatively under-researched and mostly limited to specific studies of the commissioning process. This study will include an analysis of how payers (national and local, public and private) fit with the wider ecosystem of healthcare and health research, and either facilitate or inhibit the translation of innovations into clinical practice. Our work also has implications (for example) for the regulatory processes for drugs and medical devices and the conduct of clinical trials. Therefore, we will develop Oxford BRC’s existing links to local and national policymakers and to international bodies in science and healthcare.

### Operational objectives

1. Establish overall leadership and key cross-cutting sub-themes for the ‘Partnerships’ theme.
2. Set up governance structures, including internal theme co-ordination, liaison with main BRC steering group and a ‘Partnerships’ external advisory group.
3. Gain university ethics approval for ‘research on research’ activities (i.e. those not covered by the NHS Research Ethics Committee approvals within individual research themes).
4. Establish engagement platforms and processes to strengthen partnerships with patients and the public, industry, clinical services, other elements of the NIHR research infrastructure, other academic groupings, and policymakers and payers (Fig. 3).
5. Review relevant literature on the governance and operation of health research systems.
6. Identify a sample of exemplar cases within the wider activity of the NIHR Oxford BRC, representing maximum variety in key elements of the innovation, health and wealth agenda.
7. Using case study methodology, follow and support these cases with a view to maximising value, minimising waste and extending the knowledge base.
8. For each case, evaluate progress in a way that embraces both a logic model (to what extent did they achieve what they set out to achieve?) and an evolving complex intervention in a complex system (a more nuanced narrative of what changed in each case and why).
9. Through interdisciplinary dialogue and critical reflection, produce higher-order cross-case learning as these cases unfold.
10. Feedback findings both formatively (to the individual research themes and the main BRC steering group) and summatively (as a final report).

### Over-arching research questions

1. How can we optimise a cross-cutting programme of partnership working that will support the NIHR Oxford BRC to drive health-related innovations, translate these into benefits for patients and contribute to the United Kingdom’s international competitiveness?
2. What are the generalisable lessons for the governance and cross-cutting activities of high-technology, multi-stakeholder health research systems?

For more specific research questions to be addressed by defined work packages within the ‘Partnerships’ theme, see Table 1.

**Table 1:** Data structure and analysis plan for specific research questions within the ‘Partnerships’ cross-cutting theme

Research question	Methodology	First-order data	Higher-order data
1. DRUG DEVELOPMENT. How can we improve the efficiency and patient-relevance of early-stage biotechnology drug development?	‘Open-source’ approach to early drug development and testing. Emphasis on knowledge sharing, industry collaborations and efficient harnessing and use of patient capital funds and venture capital	Narrative accounts of how drug ‘probes’ were developed, de-risked, linked with investment funding and channelled into Phase I and IIa clinical testing. Cross-case insights into blocks and bottlenecks in this pathway	Generalisable insights on how to generate the pipeline, momentum and precedent to ‘unlock’ the potential for new drug and medical device development from excellent basic science and experimental medicine
2. MEDICAL DEVICE DEVELOPMENT. How can we improve the efficiency and patient-relevance of early-stage medical device development?	Case-based interdisciplinary approach to device development that brings together clinical entrepreneurs, bioengineers, business experts and social scientists	Longitudinal narrative accounts of the unfolding fortunes of a sample of new medical devices and their inventors. Cross-case insights into technical, financial, logistical and regulatory challenges and key training needs	
3. BUSINESS DEVELOPMENT AND COMMERCIALISATION. How can we ensure that intellectual property developed as part of NHS-university partnerships is rapidly commercialised and brings prompt benefits to NHS patients?	Case-based interdisciplinary research on the fortunes of candidate innovations as they move from an abstract idea to a business case and thence to testing, trialling and scale-up	Longitudinal narrative accounts of the organisational, regulatory, political and policy challenges involved in bringing innovations to market (including the process of leveraging capital, growing a value network and tightening the value chain)	Generalisable insights on how best to support clinical entrepreneurs and how to select, de-risk and nurture innovative ideas for patient benefit in the NHS setting
4. RESEARCH METHODOLOGY, STATISTICS AND HEALTH ECONOMICS. How can we reduce waste in clinical trials of drugs, medical devices and diagnostics?	Methodological, statistical and health economics support for BRC studies from inception to ensure that clinical trials are optimally designed, the right data are collected and the best analytic and reporting methods are used	Descriptive statistics on degree of alignment between Oxford BRC’s trials and observational studies and expert standards for methodological and publication quality. Significant events.	Generalisable insights on how to improve study design, monitor and refine methodological quality as studies unfold, improve accuracy of economic modelling, and ensure consistent and high-quality reporting in publications
5. PATIENT AND PUBLIC INVOLVEMENT AND ENGAGEMENT (PPI/E) AND ETHICS. How can we achieve responsible research and innovation (reduce misalignments between entrepreneurial and commercial goals and the public good?)	Develop more efficient and scalable ways for patients and citizens to prioritise research questions and ways to follow through on such prioritisation exercises Ensure that social accountability is woven into the governance and activities of the BRC through genuine patient and citizen involvement at every level	Narrative accounts and evaluations of research prioritisation exercises; follow-up data on James Lind Alliance and similar priority-setting partnerships. Qualitative description and quantitative benchmarking of the BRC’s PPI/E activities	Generalisable insights on how to harness patient and citizen involvement in a way that ensures that innovations and research studies are desirable and aligned with the public good, and on how to measure progress towards this goal. Insights into the ethics and practicalities of managing commercial interests in the context of publicly-funded research
6. KNOWLEDGE TRANSLATION. How can we translate the findings from clinical trials rapidly and efficiently into practice?	Targeted support for BRC themes by applying the evidence base on ‘T2’ knowledge translation	Narrative accounts and evaluations of knowledge translation efforts linked to BRC biomedical themes	Generalisable insights that add to the knowledge base on research translation and implementation science
7. EDUCATION AND TRAINING. How can we best support and develop the next generation of researchers?	Ongoing training needs analysis and evaluation. Range of established courses, e.g. research design, health economics. Develop new courses, e.g. entrepreneurship, regulation, PPI/E, implementation science	Development of new courses; alignment of these with programme needs. Uptake of courses. Retention rates at every stage of early career development. Critical case studies, e.g. significant events	Generalisable insights into how to develop and support the development of individual researchers and into how education and training can efficiently and effectively build capacity for research and entrepreneurship
8. RESEARCH ON RESEARCH. What can we learn from the Oxford BRC about how best to support multi-stakeholder health research partnerships?	Targeted interview and ethnographic research on selected activities and research studies within the BRC and its linked partners	Longitudinal case narratives of particular projects or topics (research on research), incorporating both qualitative and quantitative data and applying relevant theory, e.g. from sociology and/or science and technology studies	Formative and summative learning on how to develop individuals, strengthen partnerships and maximise translational efficiency

### Study design

The study design is an organisational case study [38, 39], informed by the principles of action research [51]. We begin from the position that the knowledge base will benefit most not from a technocratic, ‘logic model’ approach to the study of innovation and research impact, but from a systematic, maximum-variety sample of richly-described and theorised case studies. As Bent Flyvbjerg has observed in his classic paper *Five Misunderstandings about Case Study Research*:

“[A] *scientific discipline without a large number of thoroughly executed case studies is a discipline without systematic production of exemplars, and … a discipline without exemplars is an ineffective one. Social science may be strengthened by the execution of more good case studies*” [38].

Given its strong track record of success against conventionally accepted metrics, NIHR Oxford BRC represents a crucial case study of a regional innovation system because it is strategically positioned to develop and test a new model of economic growth and societal impact based on high-value adding health research and the biotechnology industry. The term ‘crucial case study’ comes from the notion that, if a proposition (such as ‘synergies will result from working in cross-sector partnerships’) fails when conditions are highly favourable, then it is unlikely to work in less favourable conditions [52].

### Programme management and governance

The ‘Partnerships’ research theme is led by TG and includes a core research team (NF, PO, SS, PK, AR) whose roles include overall co-ordination and PPI/E as well as ‘research on research’. In addition to, and with a view to supporting, the main BRC steering group, a separate advisory group for the ‘Partnerships’ theme has been established, with reciprocal cross-representation. The former meets 6–8 times a year and the latter 2–3 times a year.

The ‘Partnerships’ theme has a number of sub-themes that are operationally distinct (e.g. each has a designated lead and budget), but which will work collaboratively and flexibly to develop interdisciplinary case studies centred around specific research questions (Table 1). Sub-themes include drug development, medical device development, business support and commercialisation, research methodology and statistics, health economics, bioethics, PPI/E, knowledge translation, and education and training. Sub-theme leads will meet together trimonthly; alternate sub-theme leads meetings will be oriented to producing a formal report for the external advisory group.

A wide range of external stakeholders and independent members will represent the various partnerships shown in Fig. 3 on the ‘Partnerships’ external advisory group, which will have an independent lay chair. This group will scrutinise research questions and provide critical feedback on an ongoing narrative of progress centred around exemplar interdisciplinary case studies and including both qualitative and quantitative data. Members of the external advisory group will also provide important connections to stakeholder organisations, including leveraging opportunities to increase value for money of the NIHR’s investment.

Most importantly, the external advisory group will seek to establish social accountability of the ‘Partnerships’ cross-cutting theme to patients and the public in the spirit of the ‘democratization’ of science [29, 30]. Whereas NHS-university partnerships in England are characterised by bifurcating hierarchical, legal, professional and political accountabilities to various government departments, public agencies and professional organisations, the structures and processes for establishing social accountability have yet to be formally established [6].

The ‘Partnerships’ external advisory group will address the desirability and priority of current and proposed research topics and approaches, with particular emphasis on improving patient care, educating and training tomorrow’s clinician and scientist leaders, reducing research waste and advancing translational research. An important aspect of the external advisory group’s work will be to deliberate on research priorities, such as the trade-off between research that leads to short-term patient benefits locally versus research that is more oriented to filling national or global knowledge gaps and/or to the medium or long term.

### Theoretical/conceptual framework

We will draw eclectically and critically on three closely related theoretical frameworks, which were respectively developed in innovation studies (the Triple Helix), the sociology of knowledge (Mode 2 knowledge production), and business and management (value co-creation). All have strengths and limitations and will need to be adapted as we apply them to the emerging case study. We describe these in turn.

#### Triple Helix

In their Triple Helix model, Etzkowitz et al. [53, 54] highlight how universities, industry and government, once separate and independently evolving entities, are increasingly interdependent and co-evolving, with each sector taking on elements of the others’ traditional roles (for example, universities develop the capacity to interact with industry and commercialise discoveries, industry develops the capacity to undertake research, and government provides ‘public venture capital’), while also retaining a core identity as, respectively, academic, commercial and state institutions.

Central to the Triple Helix model is the creation and support of three ‘spaces’ [55]:

- The knowledge space: collaboration to undertake research and generate new knowledge;
- The consensus space: building relationships and supporting dialogue among university, industry, healthcare staff, policymakers and citizens; and
- The innovation space: collaborations and activities to achieve the goal of implementing (and where appropriate, commercialising) research discoveries, by combining academic or technical expertise with business expertise and (public or private) venture capital.

A key component of the Triple Helix model is the notion of ‘new organisational actors’ – organisational formats that straddle traditional university–industry–state boundaries – such as incubators, science parks and new models of venture capital (including, for example, social enterprise or patient charities) [55]. Indeed, the study of the Triple Helix may focus fruitfully on the emergence and behaviour of these new organisational actors (for example, the entrepreneurial university still trains individuals in classrooms, but it also has a more contemporary role of training organisations in incubators).

Etzkowitz and Leydesdorff [53] emphasise that the Triple Helix rests on an active, questioning civil society in which public debate over values and scientific priorities, and bottom-up initiatives of various kinds, feed into the emergent decisions and actions of macro-level stakeholders. The exchange of ideas, knowledge and perspectives is facilitated by free movement of individuals between the different strands of the helix, for example, through placements for students in industry or policy and, conversely, university secondments and honorary lectureships for people from industry, government or the civil service. Such a model depends on reflexivity, that is, ongoing appraisal by the university of its evolving relationships with industry, government and civil society.

The Triple Helix metaphor is presented positively by its protagonists as enhancing the potential for innovation and economic growth in a knowledge-based society. It is worth noting that critics of the Triple Helix have warned of the danger of ‘irresponsible innovation’ that may result from a combination of strong commercial drivers, technology push, policy pull, marginalisation of ethical principles, lack of precautionary measures and tokenistic rather than democratic input from patients and citizens [56–59]. Perhaps another way of expressing this criticism is that civil society was included as part of the context for the Triple Helix but not as an equal and integral strand within it.

#### Mode 2 Knowledge Production

Our second theoretical influence is Gibbons et al.’s [60] Mode 2 Knowledge Production. In contrast to the traditional Mode 1 model of university research (produced by discipline-specific groups in ivory towers and then translated, disseminated and applied outside the academic sector), Mode 2 knowledge is “*socially distributed, application-oriented, trans-disciplinary and subject to multiple accountabilities*” [61]. As summarised previously:

“*Such knowledge is generated partly or wholly outside the university in a heterogeneous transaction space embracing university, state, economy, culture, and the wider public sphere. In this space, problems are identified, questions debated, methodologies developed, and outcomes disseminated. There are many players, many experts (of different kinds), and an evolving collective view (though rarely a consensus) on what the questions and challenges are.*” [62]

Unlike the producer-consumer or contractor-commissioner relationship between researchers and research end-users in traditional ‘Mode 1’ scientific research, relationships between researchers and users in Mode 2 are (ideally) democratic and collaborative, and seek to generate knowledge that is both socially and scientifically robust. However, such an approach constitutes a significant challenge to the norms of academic science, as set out back in 1938 by Merton [63], which include disinterestedness, objectivity and organised scepticism. The philosophical tension between a ‘disinterested’ science conducted by academics isolated from society and a ‘socially engaged’ science conducted in partnership – or at least in dialogue – with civil society is beyond the scope of this paper, but one which we will seek to explore as this programme of work unfolds.

As with the Triple Helix model, the Mode 2 model has been criticised for taking a politically naïve perspective and overlooking the power of vested interests to distort the research agenda [64, 65]. Indeed, critical academics have proposed adding Mode 0 – “*knowledge production based on relations of power and patronage*” – [66] to Gibbons et al.’s [60] original taxonomy. The question of how best to harness the co-creative potential of Mode 2 without overly distorting the research agenda with commercial and other powerful interests is one of the leading challenges of contemporary health systems research.

#### Value Co-creation

**Fig. 4:**
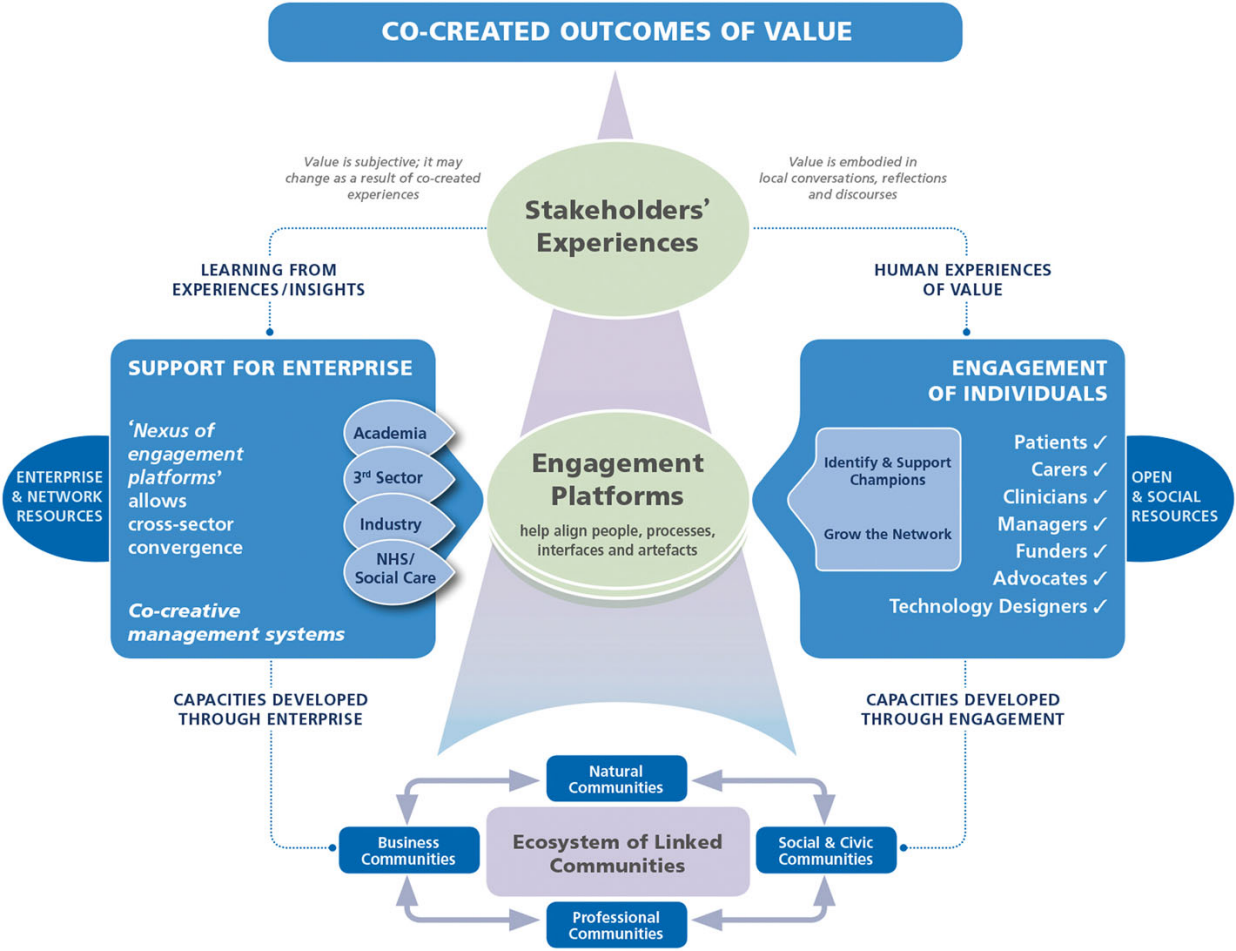
Adapted Value Co-creation model. Adapted for a public sector setting from Ramaswamy and Ozcan [67]

The third theoretical framework on which we seek to draw is an adaptation of Ramaswamy et al.’s [67, 68] Value Co-creation model – originally developed for the commercial sector as a business strategy for engaging multiple stakeholders to devise products and services to increase their value for everyone. We have adapted the Value Co-creation model to fit a setting in which the main partners are public sector health and education institutions (Fig. 4).

Ramaswamy and Ozcan [67] offer four key principles for co-creation of value across sectors:

- Stakeholders will not wholeheartedly participate in the co-creation process unless it produces value for them;
- The best way to co-create value is to focus on the experiences of all stakeholders;
- Stakeholders must be able to interact directly with one another (preferably face to face at least some of the time);
- Platforms are needed that allow stakeholders to interact and share their experiences.

Central to the model are platforms (of various kinds, formal and informal) for bringing stakeholders together. The co-creation process is supported through two key activities [67]:

- Engagement of individuals (in which people who will be key to the enterprise are engaged and offered support and open-source resources); and
- Support for enterprise, which brings organisations together to work on particular projects and programmes and provides a range of resources for this purpose.

As Fig. 4 illustrates, capacity-building occurs both through individual training and development and through the enterprise process itself. Individuals circulate within and between their various communities of practice – business, professional, natural (e.g. geographical) and civic – thus sharing experiences and perspectives. Individuals and groups value different things; communication and dialogue, including but not limited to the formal governance processes of the programme(s), ensure that each stakeholder gets something of what they value and comes to understand and contribute to what others value.

Although the Value Co-creation model originated in business studies, the theoretical ideas of co-creation can be applied to the co-creation of knowledge within the Triple Helix of university–industry–government relations resting on an active civil society [69]. To our knowledge, there has been only one published application of value co-creation in a healthcare setting (in Australia) [62].

One approach that could potentially bring all three of these perspectives together is what Carayannis et al. [70] have called “*Mode 3 knowledge production in a quadruple helix innovation system*”. This framework adds a fourth strand – civil society – to the triple helix and also extends the work of Gibbons et al. [60] to address the management of power differentials and conflicts of interest and also consider how knowledge production may occur simultaneously across Modes 1 and 2 through what Carayannis and Campbell [70] call the ‘co-evolution’ of different knowledge and innovation modes. Such an approach emphasises the value of clusters and networks that often stand in ‘co-opetition’ ([70], p. 41), involving a careful balancing of both cooperation and competition. This hybrid approach will be explored further, with pilot data, in a separate publication.

### Engagement platforms

An engagement platform has been defined by Ramaswamy and Ozcan as “*an assemblage of persons, processes, interfaces, and artifacts, whose engagement design affords environments of interactions that intensify agential actions in value creation*” ([67], p. 34).

As Fig. 4 shows, the co-creation of value depends on such platforms, which serve to bring together stakeholders from different sectors and perspectives. Engagement platforms are many and varied; they include formal governance structures, physical spaces, informal networking events and virtual interaction spaces. Examples of engagement platforms (both formal and informal) that are already in place for the Oxford BRC include (1) the inter-sectoral Strategic Partnership Board, which has a Joint Executive Group and specialist committees. These provide governance structures for the oversight of all the aspects of joint working arrangements between the Oxford University Hospitals NHS Foundation Trust and the University of Oxford in accordance with their Joint Working Agreement (Fig. 2). (2) The Oxford Academic Health Science Centre and Network, which are wider partnerships that consolidate interactions between Oxford’s universities, hospitals and industry at the local and regional levels, respectively (Fig. 1). (3) The Oxford Structural Genomics Consortium, which is a university-led, externally facing basic science research group with strong links to major pharmaceutical companies, biotechnology companies, clinical research organisations, and venture capital and patient charities. A major work stream in the ‘Partnerships’ BRC theme seeks to grow the Consortium’s industrial network and build relations with patient organisations and charities to catalyse discovery of new drug targets and inform clinical studies in ways that are patient relevant. (4) The ‘Partnerships’ external advisory group described above brings patient and lay representatives together with BRC theme leads, industry, external academics and stakeholders from the local health economy. (5) The Oxford University Clinical Academic Graduate School (OUCAGS) is a partnership between the University of Oxford and Health Education Thames Valley, which aims to support postgraduate academic education and career development for medical and non-medical staff. Via its website (www.oucags.ox.ac.uk), a regular email newsletter, a range of training courses and regular face-to-face events, it brings doctoral students and early career researchers together and links them with resources and opportunities. (6) The Oxford-BRC-Partnerships Jiscmail list is an academically run list server open to anyone working in or with the Oxford BRC, including patients and the public involved in PPI/E activities. It provides an informal platform for asynchronous email communication, interdisciplinary discussion and resource sharing on the key domains supported by the ‘Partnerships’ theme (drug and device development, industry relations and commercialisation, knowledge translation, patient and public involvement and engagement, ethics, and education and training). (7) Social media presence for the NIHR Oxford BRC includes a Twitter feed and Facebook account (@OxfordBRC). The ‘Partnerships’ theme has a dedicated ResearchGate site for publications and updates and an active Twitter feed (see ‘Planned dissemination and outputs’ below for details of these). Importantly, these social media outlets also serve as a mechanism for inviting feedback and external peer review (both academic and lay) on emerging activity.

The above list is not exhaustive. As the work of the NIHR Oxford BRC unfolds, we anticipate that the number and nature of engagement platforms will change organically. We will capture the emergence, development and attrition of key engagement platforms using ethnographic and narrative methods. Plans include the development of a single point-of-contact for BRC researchers to log help requests from the ‘Partnerships’ sub-themes (Table 1) and a series of face-to-face networking and resource-sharing events.

### Sampling of case studies

The ‘Partnerships’ theme will include a number of specific sub-themes (listed above and in Table 1), which will provide a wide variety of different kinds of support and co-ordination. Each sub-theme will select specific case studies from the NIHR Oxford BRC research themes and clusters, to which they will provide both longitudinal support and help with evaluation.

We will ensure that cases are selected to ensure maximum variety in size, structure, duration, academic discipline(s), clinical field, nature of support requested and success metrics, and that all cases have potential to contribute data to address the research questions listed above. The number of case studies will be limited by the need to provide rich description and detailed theoretical analysis (in other words, this will be a ‘small-n’ sample designed for illuminative insights and theoretical generalisability, not one designed to generate statistically representative data).

### Data collection, analysis and synthesis

The study will use a variety of qualitative and quantitative data, including the empirical studies within individual BRC research themes, narrative accounts from stakeholder interviews, ethnographic field notes, documents, and quantitative indicators and metrics of success. Data analysis and synthesis will be informed by the theoretical frameworks outlined above, and also by the objectives of action research, namely (1) informing real-time action and system change through ongoing, formative feedback, and (2) making a generalisable contribution to the knowledge base [51]; ‘generalisable’ indicates the naturalistic generalisation that is made possible through in-depth case study. In this regard, the design draws on Weick’s work on the ‘generative properties of richness’ (thick description, reflexive theorising, and ‘conceptual slack’ – openness to the many new explanations that emerge when contextual detail is added to an account) in an organisational case study [71].

Table 1 provides an overview of data structure and analysis plan for specific research questions.

### Planned dissemination and outputs

In keeping with the principles of action research and Mode 2 knowledge production, we will engage from the outset with the intended end users of our research. This will maximise the value of our research by increasing its usability and impact while reducing the need for a separate dissemination phase. Specifically, dissemination within the NIHR Oxford BRC and the broader Oxford regional innovation system will occur to a large extent through the researchers’ continuous engagement with the local and regional stakeholders and their active involvement in the co-creation of knowledge.

Dissemination beyond the Oxford regional innovation system will occur through the professional networks of the research team and the ‘Partnerships’ external advisory group, as well as through collaborations, conferences, and publications in (open-access) academic and trade journals. Patients and the public will be able to access the latest research news and multimedia from our cross-cutting theme on the dedicated webpage of the NIHR Oxford BRC (https://oxfordbrc.nihr.ac.uk/research-themes-overview/partnerships-for-health-wealth-and-innovation/). We will also use social networking and social media to increase transparency and broaden outreach. We will post regular updates on our progress and deposit our research outputs on a social networking site for scientists (https://www.researchgate.net/project/Oxford-BRC-Partnerships-for-Health-Wealth-and-Innovation). The ‘Partnerships’ theme leader (TG) is an active contributor to the social media site Twitter (@trishgreenhalgh) and will use it to reach out to global audiences in real time.

In terms of outputs, our short-term goal is to bring together university, industry and NHS partners in strategically targeted projects focused on supporting innovation and escalating promising discoveries. We will develop and strengthen links with clinical trials units and theme-specific projects to ensure that researchers receive methodological support at design stage. We will build relationships with patient/citizen groups, NHS services, industry, policymakers and payers, and undertake needs assessment for researcher training.

In the medium term, our goal is to develop a set of infrastructural capabilities to support more effective partnership dynamics; faster ‘T1’ translation of discovery science into clinical trials and observational studies; larger and more strategic collaborations with industry, the NHS and patients/citizens; research studies that meet the highest standards for methodology and value-for-money; ethical research that is valued by users; high levels of engagement with and from citizens, the local health economy, payers and national policymakers; faster ‘T2’ translation of research findings into clinical practice; research on research that feeds back into formative learning across the NIHR Oxford BRC and the wider NIHR infrastructure; and comprehensive and flexible training and career development for early-career researchers.

In the longer term, we anticipate that our work will add significant value to the NIHR Oxford BRC by unpicking the complexity of multi-stakeholder research partnerships and providing generalisable insights on how to optimise it. We predict accelerated T1 and T2 knowledge translation; closer alignment of the innovation process with patient/citizen priorities and the principles of responsible, ethical research; reduction in research waste; new knowledge about the governance and activities of multi-stakeholder research partnerships and the contexts in which they operate; and capacity-building that reflects the future needs of a rapidly-evolving health research system.

## Discussion

This study protocol has described the rationale and methodology for a novel approach to building and strengthening the various partnerships in the NIHR Oxford BRC and beyond as it embarks on its third 5-year period of NIHR funding from 2017 to 2022. We have presented our BRC as a crucial case study – a setting in which many of the preconditions for success are already in place, hence a good place to test a new approach for enhancing that success. We have introduced three linked theoretical perspectives (Triple Helix, Mode 2 Knowledge Production and Value Co-creation) relevant to the operation of large, multi-stakeholder health research partnerships. We have described the structure and governance of a new ‘Partnerships for Health, Wealth and Innovation’ cross-cutting theme, and, within that theme, we have outlined our plans to support and evaluate a maximum variety sample of cases in different aspects of partnership working. Finally, we have emphasised the importance of creating an ongoing narrative of progress to aid collective sense-making and maintain an over-arching (and evolving) vision.

Previous sociological studies of multi-stakeholder research partnerships have shown that they are inevitably characterised by structural complexity, competing interests, ambiguous loyalties and colliding institutional logics [64, 65, 72, 73]. Synergy may increase as co-governing partners work together, leading to convergence of perspectives by progressive alignment of purpose, values and goals, and growth of mutual understanding and respect. However, this is by no means inevitable; others have used the term ‘collaborative entanglement’ to depict an ongoing instability of the system that will never attain an easy synergy [74]. In some cases, organisations traditionally thought of as odd bedfellows join forces at an early stage to generate a grant application that is “*draped in the formal collaborative language and procedures prescribed by funding agency protocols*”, but in reality they continue to “*view each other pragmatically as consultants, clients or even competitors, rather than partners*” [75].

Multi-stakeholder health research partnerships have been widely studied using ethnographic case study methods [64, 65, 72–78]. However, to the best of our knowledge, this is the first study that uses linked interdisciplinary theoretical perspectives from innovation studies, sociology of science, and business and management to study multi-stakeholder partnerships in a BRC over 5 years. Another strength of this study is in using the principles of action research to inform real-time action and system change while also making a generalisable contribution to the knowledge base.

However, this study also has potential limitations. The ‘Partnerships’ theme has finite human and financial resources and it will not be logistically possible to explore all aspects of the questions listed in Table 1. Key data may not exist or may not be fully accessible to the research team. Ongoing access to undertake research on a multi-stakeholder research partnership, and real-time feedback of emerging findings in a way that shapes the work of that partnership, have both been shown to depend on the development of democratic relationships and mutual trust, which in turn stem from a smooth set-up phase and acknowledged early wins [79]. Much depends on these relational preconditions, which are highly contingent and cannot be achieved purely by establishing the “right” governance structures [78].

It is also important to note that the study design is intentionally a single (‘n of 1’) large case study containing multiple (‘small n’) focused case studies, selected for their contrasting features. This model, favoured by leading organisational scholars as the most appropriate one for studying complex systems [38, 71], is designed to generate illuminative insights and naturalistic generalisability through the use of thick description and reflexive theorising. The study design will not generate statistically representative data, nor is it primarily intended to produce cross-case theoretical insights (as would occur, for example, in a small-n realist evaluation). In other words, we seek to answer the important and context-bound question ‘what is going on here and what can we learn from it?’, not ‘what is the effect size?’ or ‘what works for whom under different circumstances?’

The single case study design has been chosen because the NIHR Oxford BRC is a unique, dynamic and continuously evolving research system that will be influenced by future research policy developments and organisational changes. As we write this, the United Kingdom faces a changing context for scientific research, drug regulation and health services delivery as a result of its decision to leave the European Union. The unfolding of research partnerships will need to be carefully analysed with relation to what may turn out to be dramatic changes in their external context. We believe the use of the single case study for such a study is amply justified on theoretical grounds and have provided detailed philosophical explanation elsewhere [37, 80]. However, the n of 1 organisational case study is not well understood by many in the biomedical research field. There is, therefore, a risk that our findings will lack credibility in the eyes of those who value a more experimental approach and expect a ‘control group’.

In sum, this paper has described the rationale, aims, objectives and methodology for an ambitious programme of work to both support and systematically study the work of one of the largest and – currently – most successful of the NIHR’s BRCs as it enters its third 5-year funding period. The case study appears to contain many of the key ingredients for success, but there are also many unknowns, finite resources and an unstable external context. We anticipate that our methodology will, at the very least, produce a meaningful narrative in 5 years’ time of how the NIHR Oxford BRC’s fortunes unfolded and why.

## Abbreviations

AHSC: Academic Health Sciences Centre
BRC: Biomedical Research Centre
CLAHRC: Collaboration for Leadership in Applied Health Research and Care
NHS: National Health Service
NIHR: National Institute for Health Research
PPI/E: Patient and public involvement and engagement

## References

[1] Rycroft-Malone J, Burton C, Wilkinson JE, Harvey G, McCormack B, Baker R (2015). Collective action for knowledge mobilisation: a realist evaluation of the Collaborations for Leadership in Applied Health Research and Care. Health Services and Delivery Research.

[2] Snape K, Trembath R, Lord G (2008). Translational medicine and the NIHR Biomedical Research Centre concept. QJM.

[3] National Institute for Health Research. Biomedical Research Centres. http://www.nihr.ac.uk/about-us/how-we-are-managed/our-structure/infrastructure/biomedical-research-centres.htm. Accessed 3 March 2017.

[4] Walshe K, Davies HT (2013). Health research, development and innovation in England from 1988 to 2013: from research production to knowledge mobilization. J Health Serv Res Policy.

[5] Ovseiko PV, Davies SM, Buchan AM (2010). Organizational models of emerging academic health science centers in England. Acad Med.

[6] Ovseiko PV, Heitmueller A, Allen P, Davies SM, Wells G, Ford GA (2014). Improving accountability through alignment: the role of academic health science centres and networks in England. BMC Health Serv Res..

[7] Ovseiko PV, O'Sullivan C, Powell SC, Davies SM, Buchan AM (2014). Implementation of collaborative governance in cross-sector innovation and education networks: evidence from the National Health Service in England. BMC Health Serv Res..

[8] McGough R, Rubenstein S (2013). Academia. Shaping the new science networks. Health Serv J.

[9] Department of Health. Best Research for Best Health: A New National Health Research Strategy. https://www.gov.uk/government/publications/best-research-for-best-health-a-new-national-health-research-strategy. Accessed 29 June 2017.

[10] Woolf SH (2008). The meaning of translational research and why it matters. JAMA.

[11] Shahzad A, McLachlan CS, Gault J, Cohrs RJ, Wang X, Köhler G. Global translational medicine initiatives and programs. Translational Biomedicine. 2011;2(3):2.

[12] Mittra J (2015). The New Health Bioeconomy: R&D Policy and Innovation for the Twenty-first Century.

[13] Marjanovic S, Soper B, Shehabi A, Celia C, Reding A, Ling T. Changing the translational research landscape: A review of the impacts of Biomedical Research Centres in England. https://www.rand.org/pubs/technical_reports/TR787.html. Accessed 29 June 2017. 10.7249/rhq-01-04-12 PMC4945261 28083219

[14] Lichten CA, Marsden G, Pollitt A, Kiparoglou V, Channon KM, Sussex J (2017). Does a biomedical research centre affect patient care in local hospitals?. Health Res Policy Syst..

[15] Hampson G, Lichten C, Berdud M, Pollitt A, Mestre-Ferrandiz J, Sussex J, et al. ‘Macro’ Evaluation of the NIHR Oxford Biomedical Research Centre. https://www.ohe.org/publications/%E2%80%98macro%E2%80%99-evaluation-nihr-oxford-biomedical-research-centre-0. Accessed 29 June 2017.

[16] Bienkowska-Gibbs T, Exley J, Saunders CL, Marjanovic S, Chataway J, MacLure C (2016). Evaluating the role and contribution of innovation to health and wealth in the UK: a review of innovation, health and wealth: phase 1 final report. Rand Health Q.

[17] Naylor D, Fraser N, Girard F, Jenkins T, Mintz J, Power C. Unleashing innovation: Excellent healthcare for Canada. Report of the Advisory Panel on Healthcare Innovation. http://www.healthycanadians.gc.ca/publications/health-system-systeme-sante/report-healthcare-innovation-rapport-soins/alt/report-healthcare-innovation-rapport-soins-eng.pdf. Accessed 26 February 2017.

[18] Walshe K, McKee M, McCarthy M, Groenewegen P, Hansen J, Figueras J (2013). Health systems and policy research in Europe: Horizon 2020. Lancet.

[19] Chalmers I, Bracken MB, Djulbegovic B, Garattini S, Grant J, Gülmezoglu AM (2014). How to increase value and reduce waste when research priorities are set. Lancet.

[20] Lehoux P, Daudelin G, Williams-Jones B, Denis J-L, Longo C (2014). How do business model and health technology design influence each other? Insights from a longitudinal case study of three academic spin-offs. Res Policy.

[21] Ferlie E, Crilly T, Jashapara A, Peckham A (2012). Knowledge mobilisation in healthcare: a critical review of health sector and generic management literature. Soc Sci Med.

[22] Hanney SR, González-Block MA (2016). Building health research systems: WHO is generating global perspectives, and who’s celebrating national successes?. Health Res Policy Syst..

[23] Hernandez-Villafuerte K, Sussex J, Robin E, Guthrie S, Wooding S (2017). Economies of scale and scope in publicly funded biomedical and health research: evidence from the literature. Health Res Policy Syst..

[24] Ferlie E, Fitzgerald L, McGivern G, Dopson S, Bennett C (2013). Making Wicked Problems Governable?: the case of managed networks in health care.

[25] Department of Health. Innovation, Health and Wealth: Accelerating Adoption and Diffusion in the NHS. http://webarchive.nationalarchives.gov.uk/20130107105354/http:/www.dh.gov.uk/en/Publicationsandstatistics/Publications/PublicationsPolicyAndGuidance/DH_131299. Accessed 29 June 2017.

[26] Smith J. Technological Innovation in Health Care: Report of the Standing Commission on Health. http://www.ourcommons.ca/DocumentViewer/en/41-1/HESA/report-14. Accessed 26 February 2017.

[27] Garber S, Gates S, Keeler EB, Valana ME, Mulcahy AW, Lau C, et al. Redirecting Innovation in U.S. Health Care: Options to Decrease Spending and Increase Value. http://www.rand.org/pubs/research_reports/RR308.html. Accessed 27 February 2017. 10.7249/rhq-04-01-03 PMC5051971 28083317

[28] National Institute for Health Research. Going the Extra Mile: Improving the Nation’s Health and Wellbeing through Public Involvement in Research. https://www.rds-yh.nihr.ac.uk/wp-content/uploads/2015/06/Going-the-Extra-Mile-Final.pdf. Accessed 3 Mar 2017.

[29] Nowotny H, Scott P, Gibbons M (2001). Re-thinking Science: Knowledge and the Public in an Age of Uncertainty.

[30] Kleinman DL, Kleinman DL (2000). Democratizations of Science and Technology. Science, Technology, and Democracy.

[31] Brett J, Staniszewska S, Mockford C, Herron-Marx S, Tysall C, Hughes J (2014). A systematic review of the impact of patient and public involvement on service users, researchers and communities. The patient—Patient Centred Outcomes Research. Health Expect.

[32] Edelman N, Barron D (2015). Evaluation of public involvement in research: time for a major re-think?. J Health Serv Res Policy.

[33] Gradinger F, Britten N, Wyatt K, Froggatt K, Gibson A, Jacoby A (2015). Values associated with public involvement in health and social care research: a narrative review. Health Expect.

[34] Greenhalgh T, Shaw S, Fahy N (2017). The bright elusive butterfly of value in health technology development. Int J Health Policy Management.

[35] Boaz A, Biri D, McKevitt C (2014). Rethinking the relationship between science and society: Has there been a shift in attitudes to Patient and Public Involvement and Public Engagement in Science in the United Kingdom?. Health Expect.

[36] Munafò MR, Nosek BA, Bishop DV, Button KS, Chambers CD, du Sert NP (2017). A manifesto for reproducible science. Nat Human Behav..

[37] Russell J, Greenhalgh T, Kushner S, Russell J, Greenhalgh T, Kushner S (2015). Case study evaluation: Past, present and future challenges. Case Study Evaluation: Past, Present and Future Challenges (Advances in Program Evaluation, Volume 15).

[38] Flyvbjerg B (2006). Five misunderstandings about case-study research. Qual Inq.

[39] Eisenhardt KM (1989). Building theories from case study research. Acad Manag Rev.

[40] Nosek BA, Alter G, Banks GC, Borsboom D, Bowman S, Breckler S (2015). Promoting an open research culture. Science.

[41] Boaz A, Fitzpatrick S, Shaw B (2009). Assessing the impact of research on policy: A literature review. Sci Public Policy.

[42] Oxfordshire Local Enterprise Partnership. The Oxfordshire Innovation Engine: realising the growth potential. http://www.sqw.co.uk/files/2613/8690/7243/Oxford_engine.pdf. Accessed 3 March 2017.

[43] Oxfordshire Local Enterprise Partnership. The Oxfordshire Innovation Engine Update http://www.oxfordahsn.org/wp-content/uploads/2016/07/Oxfordshire-Innovation-Engine-Update-2016-FINAL-REPORT-2.pdf. Accessed 3 March 2017.

[44] Ovseiko PV, Oancea A, Buchan AM (2012). Assessing research impact in academic clinical medicine: a study using Research Excellence Framework pilot impact indicators. BMC Health Serv Res..

[45] Ovseiko PV, Davies SM, Buchan AM (2014). Funding of academic research in clinical medicine in the United Kingdom. Acad Med.

[46] Independent Commission on Health Inequalities in Oxfordshire. Addressing Health Inequalities in Oxfordshire. http://www.healthwatchoxfordshire.co.uk/sites/default/files/health_inequalities_headline_report.pdf. Accessed 3 Mar 2017.

[47] Greenhalgh T (2018). How to implement evidence-based healthcare.

[48] Bauer MS, Damschroder L, Hagedorn H, Smith J, Kilbourne AM (2015). An introduction to implementation science for the non-specialist. BMC Psychol..

[49] Holmes BJ, Best A, Davies H, Hunter D, Kelly MP, Marshall M, et al. Mobilising knowledge in complex health systems: a call to action. Evid Policy J Res Debate Pract. 2016. https://doi.org/10.1332/174426416X14712553750311.

[50] Open for Innovation: UK Biopharma R&D Sourcebook. http://www.abpi.org.uk/our-work/library/industry/Pages/Open-for-Innovation-ABPI-Sourcebook-2016.aspx. Accessed 7 Apr 2017.

[51] Waterman H, Tillen D, Dickson R, De Koning K (2001). Action research: a systematic review and guidance for assessment. Health Technol Assess.

[52] Eckstein H, Greenstein FI, Polsby NW (1975). Case study and theory in political science. Handbook of Political Science.

[53] Etzkowitz H, Leydesdorff L (2000). The dynamics of innovation: from National Systems and “Mode 2” to a Triple Helix of university–industry–government relations. Res Policy.

[54] Ranga M, Etzkowitz H (2013). Triple helix systems: an analytical framework for innovation policy and practice in the Knowledge Society. Ind High Educ.

[55] Etzkowitz H (2008). The triple helix: university-industry-government innovation in action.

[56] Grunwald A (2011). Responsible innovation: bringing together technology assessment, applied ethics, and STS research. Enterprise Work Innovation Stud..

[57] Von Schomberg R, Owen R, Heintz M, Bessant J (2013). A vision of responsible research and innovation. Responsible Innovation: Managing the Responsible Emergence of Science and Innovation in Society.

[58] Owen R, Macnaghten P, Stilgoe J (2012). Responsible research and innovation: From science in society to science for society, with society. Sci Public Policy.

[59] Stilgoe J, Owen R, Macnaghten P (2013). Developing a framework for responsible innovation. Res Policy.

[60] Gibbons M, Limoges C, Nowotny H, Schwartzman S, Scott P, Trow M (1994). The new production of knowledge: The dynamics of science and research in contemporary societies.

[61] Nowotny H, Scott P, Gibbons M (2003). Mode 2 revisited: The new production of knowledge. Minerva.

[62] Greenhalgh T, Jackson C, Shaw S, Janaiman T (2016). Achieving research impact through co-creation in community-based health services: literature review and case study. Milbank Q.

[63] Merton RK (1938). Science and the social order. Philos Sci.

[64] Swan J, Bresnen M, Robertson M, Newell S, Dopson S (2010). When policy meets practice: Colliding logics and the challenges of ‘Mode 2’initiatives in the translation of academic knowledge. Organ Stud.

[65] Orr K, Bennett M (2012). Public administration scholarship and the politics of coproducing academic–practitioner research. Public Adm Rev.

[66] Bresnen M, Burrell G (2013). Journals à la mode? Twenty years of living alongside Mode 2 and the new production of knowledge. Organization.

[67] Ramaswamy V, Ozcan K (2014). The co-creation paradigm.

[68] Ramaswamy V, Gouillart FJ (2010). The power of co-creation: Build it with them to boost growth, productivity, and profits.

[69] Hughes T (2014). Co-creation: moving towards a framework for creating innovation in the Triple Helix. Prometheus.

[70] Carayannis EG, Campbell DF (2012). Mode 3 knowledge production in quadruple helix innovation systems. Mode 3 Knowledge Production in Quadruple Helix Innovation Systems.

[71] Weick KE (2007). The generative properties of richness. Acad Manag J.

[72] Wehrens R, Bekker M, Bal R (2014). Hybrid management configurations in joint research. Sci Technol Hum Values.

[73] Hanney S, Kuruvilla S, Soper B, Mays N (2010). Who needs what from a national health research system: lessons from reforms to the English Department of Health's R&D system. Health Res Policy Syst..

[74] Bennet A, Bennet D, Fafard K, Fonda M, Lomond T, Messier L (2007). Knowledge Mobilization in the Social Sciences and Humanities.

[75] Hinchcliff R, Greenfield D, Braithwaite J (2014). Is it worth engaging in multi-stakeholder health services research collaborations? Reflections on key benefits, challenges and enabling mechanisms. Int J Qual Health Care.

[76] Fitzgerald L, Harvey G (2015). Translational networks in healthcare? Evidence on the design and initiation of organizational networks for knowledge mobilization. Soc Sci Med..

[77] Brown C (2012). The policy agora: how the epistemological and ideological preferences of policy-makers affect the development of government policy. Human Welfare.

[78] Schmachtel S (2015). Local partnerships as ‘rationalized myths’: a critical examination of the micro-discourse in educational partnership working. Crit Policy Stud.

[79] Jagosh J, Bush PL, Salsberg J, Macaulay AC, Greenhalgh T, Wong G (2015). A realist evaluation of community-based participatory research: partnership synergy, trust building and related ripple effects. BMC Public Health..

[80] Greenhalgh T, Russell J, Ashcroft RE, Parsons W (2011). Why national eHealth programs need dead philosophers: Wittgensteinian reflections on policymakers' reluctance to learn from history. Milbank Q.

